# Prevalence of Methicillin-Resistant *Staphylococcus aureus* in Shrines

**DOI:** 10.1155/2020/7981648

**Published:** 2020-02-29

**Authors:** Charu Arjyal, Jyoti KC, Shreya Neupane

**Affiliations:** Tri-Chandra Multiple Campus, Tribhuvan University, Kathmandu, Nepal

## Abstract

Methicillin-resistant *Staphylococcus aureus* (MRSA) infection in human beings and animals is concerning; it stands out as one of the leading agents causing nosocomial and community infections. Also, marginally increasing drug resistance in MRSA has limited therapeutic options. This study focuses on estimating the prevalence of MRSA in shrines, a place where human and animal interaction is frequent, sharing antibiotic-resistant bacteria, antibiotic-resistant genes, and diseases. A total of 120 environmental swabs were collected from targeted areas during the study period, March 2018 to May 2018. *Staphylococcus aureus* was identified by growth on mannitol salt agar (MSA), and MRSA by growth on mannitol salt agar containing 4 *μ*g Oxacillin, Gram staining, and conventional biochemical test. Isolates of *S. aureus* were characterized by antibiotic susceptibility testing using the disc diffusion method. MRSA and methicillin-sensitive *S. aureus* (MSSA) proportion were 19% and 81%, respectively; a high rate of MRSA was observed in isolates from Thapathali (28.6%). MSSA isolates showed a high rate of resistance to erythromycin (64.7%). MRSA isolates were resistant to gentamicin (50%), cotrimoxazole (25%), erythromycin (50%), and ciprofloxacin (25%). The isolates were susceptible to linezolid (100%), clindamycin (100%), ciprofloxacin (75%), erythromycin (50%), tetracycline (100%), and cotrimoxazole (75%). Intermediate resistance was also found in gentamicin (50%). Of the 11 MSSA isolates that were erythromycin resistant and clindamycin sensitive, 6 (54.5%) showed the inducible clindamycin resistance (ICR) pattern and 2 MRSA isolates that were erythromycin resistant and clindamycin sensitive showed ICR pattern. Fifteen MSSA isolates were *β*-lactamase positive, whereas only two MRSA isolates showed *β*-lactamase production. There exists a minimal research work on infectious diseases that are shared between primates and animals. This study suggests the pervasiveness of MRSA/MSSA in the shrines, which may be a primary place for pathogen exchange between humans and primates.

## 1. Introduction

*Staphylococcus aureus* (*S*. *aureus*) has been continuously evolving and developing resistance to antibiotics since the medical use of penicillin began in 1942 [1]. Penicillin works by inhibiting penicillin-binding protein (PBP), which is crucial in cell wall synthesis of bacteria. The inhibition of PBP means the bacteria die from osmosis [2]. Bacteria soon began producing penicillinase enzyme, a specific type of *ß*-lactamase, which hydrolyzes the antibiotic and makes it ineffective. This production led to the introduction of semisynthetic penicillinase-resistant penicillin called methicillin. The working mechanism of methicillin is similar to that of penicillin with the difference of extra methoxy group that produces an enzyme that reduces the affinity for staphylococcal ß-lactamase [3]. Shortly, however, *Staphylococcus aureus* started exhibiting resistance to methicillin treatment. These resistant strains emerged in the United Kingdom [4] and became known as methicillin-resistant *S. aureus* (MRSA).

MRSA has become a prime nosocomial pathogen for patients in hospitals and nursing homes during the past ten years [5–8]. Moreover, community-acquired MRSA (CA-MRSA) infections are on the rise; the transmission of MRSA from the community beyond acute care hospital environments is receiving more attention [9–11]. Evidence suggests that contact with contaminated environmental surfaces is a significant transmission factor [12–16]. Outbreaks of CA-MRSA have occurred among individuals sharing close contact with others in schools, prisons, and locker rooms. Still, other possible environmental reservoirs of MRSA have yet to be comprehensively explored [17, 18]. Similarly, little is known about *S. aureus* or MRSA colonization and infection in nonhuman primates, which are essential research models for human disease [19–22].

Shrines can be considered a central location for the association of primates and people. There occurs a close contact between them as humans, both intentionally and unintentionally, feed the resident primates. Such close contact forms the basis for the transmission of infections, including MRSA. It is known that captured primates can acquire tuberculosis and MRSA from their human caregivers, but it is unclear in what conditions wild free-ranging primates both provide and receive pathogenic bacteria from human interaction [23]. Most of the studies done till today are confined in the hospital settings because of which other areas like CA-MRSA are in shadow. This may have been because of the immediate threat that hospital-acquired MRSA (HA- MRSA) brings to the population. Hence, this study can bring light to the condition of MRSA in shrines, which can be considered a perfect setting for the transfer of MRSA between humans and primates.

## 2. Materials and Methods

### 2.1. Materials

The materials, equipment, and various reagents used in different stages of this study are listed in Appendix A.

### 2.2. Methods

#### 2.2.1. Research Method

The research method was quantitative, and primary data were collected from March 2018 to May 2018 from shrines inside Kathmandu valley. Samples were processed in the laboratory of Nepalese Farming Institute, Maitidevi, Kathmandu.

#### 2.2.2. Study Variables

The variables of the study were an occurrence of *S. aureus*, MRSA, different shrines, and antibiotic susceptibility profile.

#### 2.2.3. Research Design

The study was field-based and cross-sectional.

#### 2.2.4. Study Site and Its Justification

The study was carried out in six shrines located at Kathmandu and Bhaktapur cities of Kathmandu valley.

No information is available about *S. aureus*/MRSA prevalence in shrines; hence, this study site has been selected for research.

#### 2.2.5. Sample Size

A total of 120 samples (environmental swabs) were collected from the shrine area.

#### 2.2.6. Data Collection Techniques/Methods

*(1) Specimen Collection and Transport*. Using a sterile environmental swab (sponge swabs) moistened with buffered peptone broth, several surfaces around shrines frequently visited by humans and monkeys were gently swabbed. The collected swabs were kept in the vial, screw-capped, clearly labelled, and transported to the laboratory immediately to avoid contamination.

*(2) Specimen Processing*.

**Figure 1:**
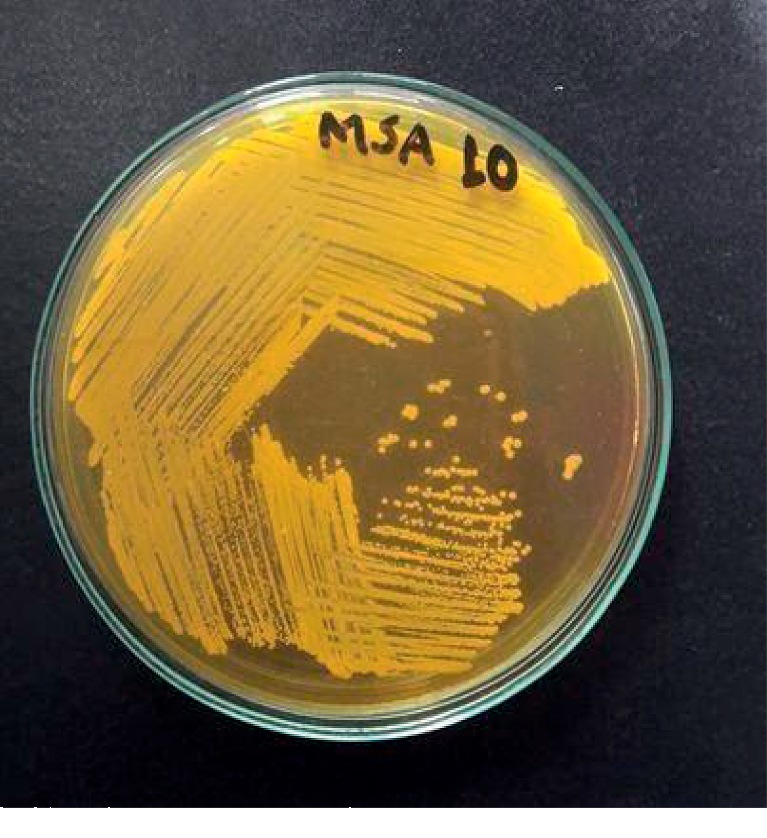
Growth of *S. aureus* on mannitol salt agar (yellow colonies after 24 hours incubation at 37°C) (isolate no. 10).

**Figure 2:**
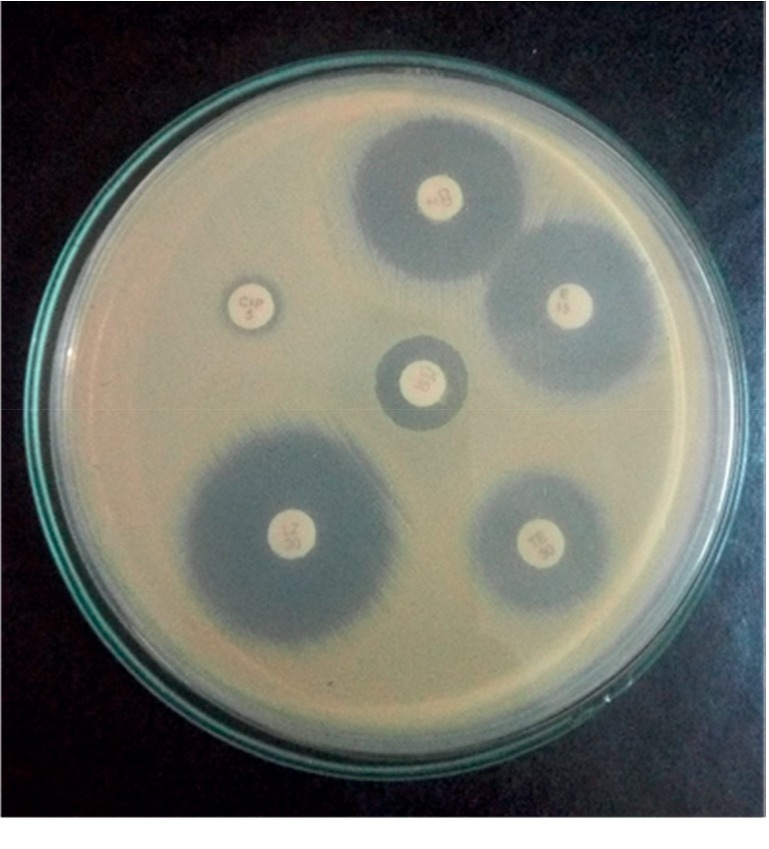
Antibiotic susceptibility pattern of MRSA.

**Figure 3:**
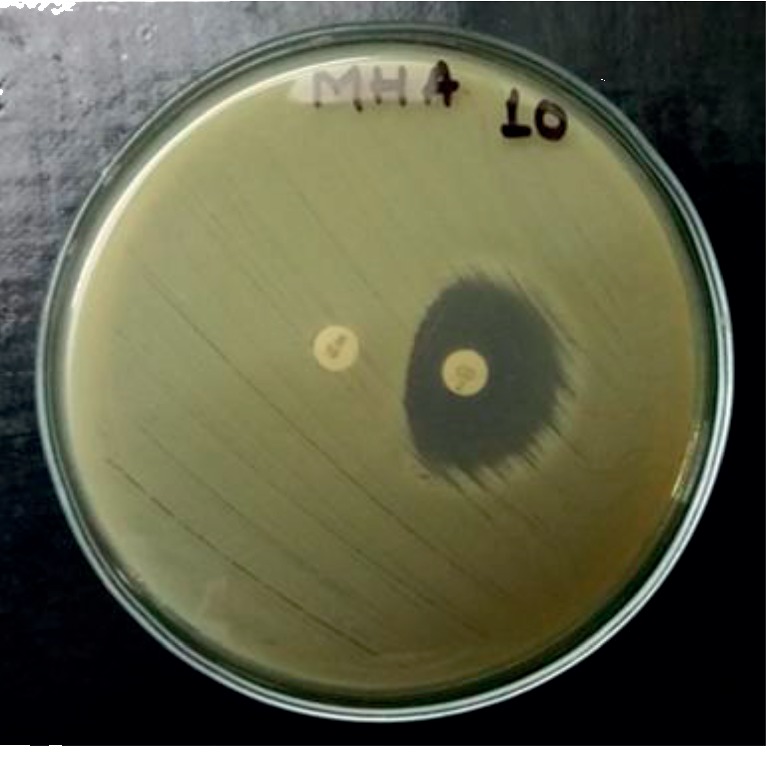
Inducible clindamycin resistant (D-test) (isolate no. 10).

**Figure 4:**
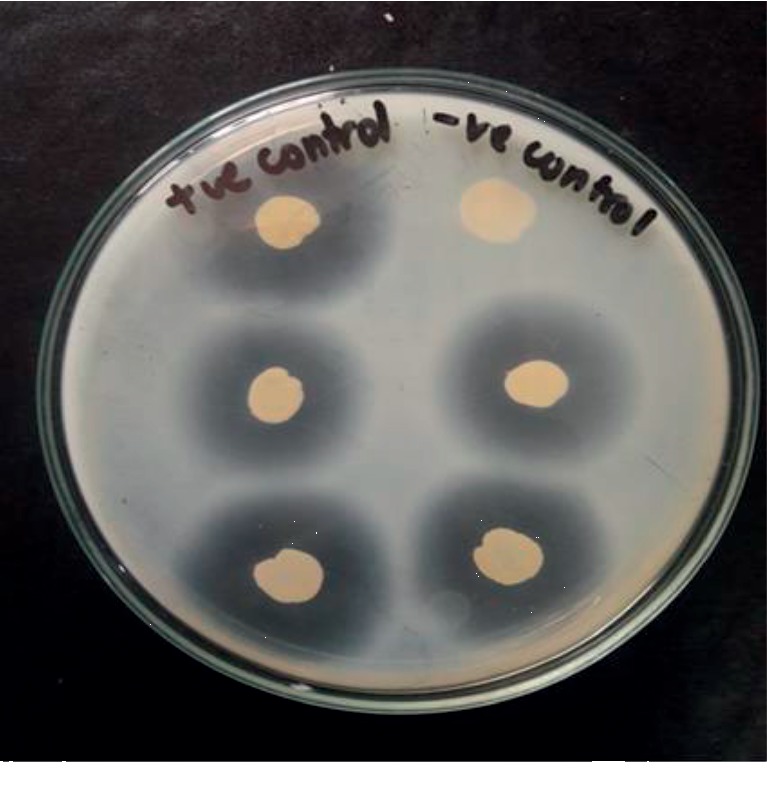
*S. aureus* on DNase agar.

- Isolation of *S. aureus*
- The environment swabs were enriched in M-Staph broth and incubated for 48 hours in anaerobic conditions. The black precipitate obtained was directly inoculated in MSA agar for 24 hours. Mannitol fermenting colonies (yellow colonies) from MSA were subcultured on nutrient agar and incubated at 37°C for 24 hours. Golden yellow colonies on nutrient agar having round, convex, opaque, and smooth-glistening surface with a diameter of about 2–3 mm were indicative of *S. aureus.* Further phenotypic identification of the *S. aureus* was made by Gram staining, catalase test, oxidase test, coagulase test (slide and tube test), and oxidative/fermentative test (Figure 1).
- Detection of MRSA
- The MRSA isolates were identified by growth on mannitol salt Agar (MSA) containing 4 *μ*g/ml oxacillin (CLSI 2014). Four oxacillin-resistant *S. aureus* isolates were tested for the *mecA* gene–a molecular marker of methicillin resistance in *S. aureus* by modified Kirby-Bauer disc diffusion method using cefoxitin (30 *μ*g) disc (CLSI 2014). Isolates resistant to cefoxitin were noted as MRSA and susceptible one as MSSA.
- Antibiotic susceptibility testing by disc diffusion method
- All identified MRSA isolates were subjected to in vitro antibiotic susceptibility tests by the modified Kirby-Bauer disc diffusion method recommended by CLSI guidelines (CLSI 2014). The antibiotics tested were gentamicin (10 *μ*g), erythromycin (15 *μ*g), ciprofloxacin (5 *μ*g), tetracycline (30 *μ*g), clindamycin (2 *μ*g), cotrimoxazole (1.25/23.75 *μ*g), and linezolid (30 *μ*g). Briefly, the inoculums were prepared by transferring 3–4 identical colonies from the nutrient agar to sterile normal saline. The turbidity of the inoculums was made equivalent to a 0.5 McFarland standard. The lawn culture of the test inoculums was prepared by swabbing Mueller-Hinton agar (MHA) with a sterile cotton swab dipped into inoculums. Antibiotic discs were applied to the inoculated MHA plate and incubated at 37°C for 18 hours. After incubation, the zone of inhibition around the discs was noted, and the results were interpreted as sensitive, intermediate, or resistant (CLSI 2014) (Figure 2).
- Detection of inducible clindamycin resistance in *S. aureus*
- For *S. aureus* that was erythromycin (15 *μ*g) resistant and Clindamycin (2 *μ*g) sensitive, the D-zone test was performed to detect inducible clindamycin resistance. In the lawn culture of test inoculums on MHA, erythromycin and clindamycin were placed 15–26 mm apart and incubated at 37°C for 18 hours. After incubation, the flattening of the clindamycin zone of inhibition adjacent to the erythromycin disc (referred to as a D-zone) was indicative of inducible clindamycin resistance (Figure 3).
- Detection of *β*-lactamase
- *β*-Lactamase production was detected by the penicillin disc diffusion zone-edge test recommended by CLSI (2014). The turbidity of the inoculum was made equivalent to a 0.5 McFarland standard. The lawn culture of the test inoculums was prepared by swabbing MHA with a sterile cotton swab dipped into inoculums. The penicillin (10 *μ*g) disc was used for the detection of *β*-lactamase production.
- Detection of DNase
- DNase test was performed to determine the ability of an organism to produce the DNase enzyme, presumptively to differentiate *Staphylococcus aureus* that produces the enzyme deoxyribonuclease from other Staphylococci which do not produce deoxyribonuclease. The test organism was inoculated onto a small area of the DNase test agar plate, which was then incubated at 37°C for 24 hours. After incubation, the surface of agar was flooded with 1 N HCL solution (Figure 4).

## 3. Results

### 3.1. Occurrence of MRSA/MSSA in the Environmental Samples

**Figure 5:**
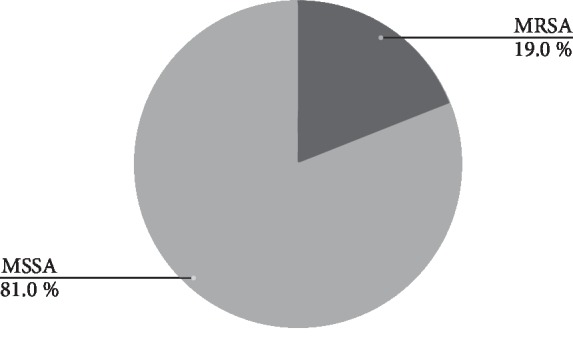
Occurrence of MRSA and MSSA in the environment sample.

Out of 120 environmental samples collected from 6 different shrines located in the Kathmandu valley, a total of 21 *S. aureus* were isolated (17.5%); 4 isolates exhibited methicillin resistance (19%) (MRSA), and 17 isolates were methicillin susceptible (81%), as shown in Figure 5.

### 3.2. Distribution of *S. aureus* among Different Sites

Of all MRSA isolates, the highest number of *S. aureus* was observed in environmental samples from Thapathali (7), followed by Pashupati (4), Nilbarahi (4), Swayambhu (3), and Guheshwori (2). The sample collected from Bajrayogini exhibited low *S. aureus* occurrence (1), as shown in Table 1.

### 3.3. Distribution of MRSA among Different Sites

Out of 21 *S. aureus* isolated, 4 were MRSA. The highest number of MRSA was isolated from Thapathali (2). One isolate was isolated from Pashupati and Nilbarahi. No MRSA was detected in Swayambhu, Bajrayogini, and Guheshwori (see Table 2).

### 3.4. Antibiotic Susceptibility Pattern of MSSA

Among the MSSA isolates, highest resistance to erythromycin (*n* = 11; 64.7%) was observed, followed by ciprofloxacin (*n* = 8; 47.0%), cotrimoxazole (*n* = 4; 23.5%), and gentamicin (*n* = 1; 5.8%). All of the isolates were susceptible to linezolid, tetracycline, and clindamycin (see Table 3).

### 3.5. Antibiotic Susceptibility Pattern of MRSA

Among the MRSA isolates, the highest resistance to erythromycin and gentamicin (*n* = 2; 50%) was observed, followed by ciprofloxacin (*n* = 1; 25%) and cotrimoxazole (*n* = 1; 25%), as shown in Table 4. All of the isolates were susceptible to linezolid, tetracycline, and clindamycin.

### 3.6. Inducible Clindamycin Resistance in MSSA and MRSA

**Figure 6:**
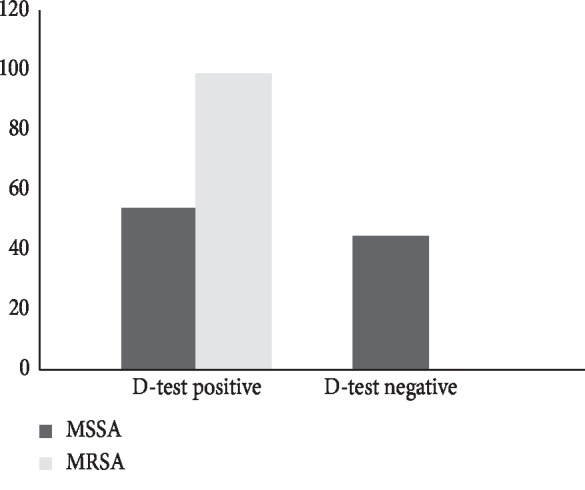
Inducible clindamycin resistance in MSSA and MRSA.

Of the total 11 MSSA isolates that were erythromycin resistant and clindamycin sensitive, 6 (54.5%) showed the inducible clindamycin resistance pattern and 5 (45.5%) showed negatively inducible clindamycin resistance, whereas out of 2 MRSA that met the criteria, both were D-test positive (see Figure 6).

### 3.7. *β*-Lactamase Production among MSSA and MRSA

**Figure 7:**
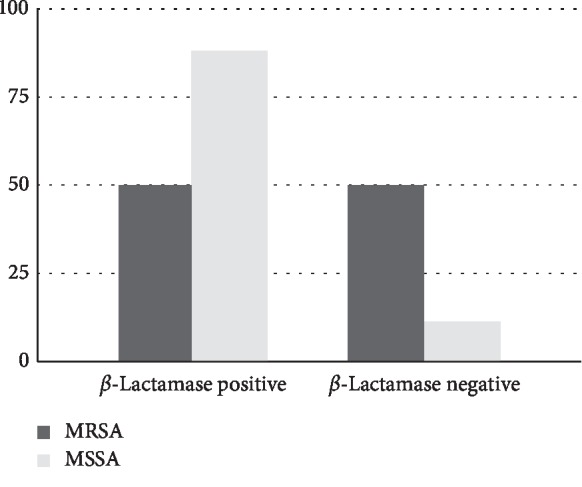
*β*-Lactamase production among MSSA and MRSA.

Among 17 MSSA isolates, 15 (88%) were *β*-lactamase positive, whereas of 4 MRSA, only 2(50%) showed *β*-lactamase production (see Figure 7).

## 4. Discussion

Diversified MRSA epidemiology is considered a significant health concern in clinical and community settings. Studies conducted to date have reported a high prevalence of MRSA colonization in hospital settings than community environments; however, no clear distinction criteria are present for the detection of MRSA origin. Furthermore, relatively little is known about the dynamics of *S. aureus* or MRSA colonization in nonhuman primates, which are essential research models for human disease [19, 21, 22, 24].

Shrines, for the most part, are rife with primates. As people place offerings on a votive altar, they come in contact with the surface previously touched by primates or the primates in the hope of finding food, and roam in the vicinity making each susceptible to the transmission of infection. We characterized MRSA in environmental samples from such areas to predict the distribution of MRSA. This study has generated data to evaluate the condition of MRSA in shrines. A total of 120 environmental samples were collected from 6 shrine areas of Kathmandu.

The screening of environmental samples revealed that the carriage rate of *S. aureus* was 17.5% and of MRSA 19%, comparatively higher than the study conducted near temples areas in Kathmandu [23], where 59 saliva samples were collected from wild monkeys (*Macaca mulatta*), among which 6.8% macaques MRSA were isolated, with 3 ST22 SCCmec type IV and one ST239 type III; this being the first isolation of MRSA ST22 SCCmec IV from primates. A study conducted by Mbogori et al. in Nairobi County from 306 samples of toilet and classroom door handles collected using sterile swabs reported the prevalence of *S. aureus* as 20% and 15% as MRSA positive [25]; a slightly high MRSA rate was observed in our study. The variance in the rate of MRSA in these studies might have been due to the difference in sample numbers and the area from which samples were obtained.

A relatively high number of MRSA was isolated from Thapathali (*n* = 2; 28.6%), whereas 3 shrines showed no occurrence of MRSA. This could be due to the close contact primates in Thapathali shared with the residents. The MRSA carriage depends on frequent contact with personnel's inanimate objects in the environment. There is limited research on whether primates receive pathogenic bacteria from human interaction or whether human beings can become the host for organisms transferred from these primates at shrines and parks. This inadequacy hampers our ability to compare the rates of transfer of bacteria.

MRSA showed high resistance to erythromycin and gentamicin (50%). Resistance towards ciprofloxacin and cotrimoxazole was moderate (25%). Correspondingly low resistance pattern of gentamicin (27.7%) was reported in CA-MRSA isolated from shrine areas in a study conducted by Al-Mohana et al., but the same study showed a high resistance pattern of erythromycin (72.7%) and ciprofloxacin (45.4%) [26]. Drugs that are highly preferred may result in resistance and are mediated by the acquisition of the genes that confer resistance to such antibiotics. A similar suggestion was given by Jaimes et al. that the development of antimicrobial resistance is nearly always as a repeated therapeutic or indiscriminate use of them [27].

The intermediate resistance was found against gentamicin (50%). Aminoglycoside antibiotics such as kanamycin, gentamicin, and streptomycin were effective against staphylococcal infections until strains evolved mechanisms to inhibit the aminoglycosides action—inhibition of the initiation complex formation and misreading of the mRNA.

The MRSA strains were susceptible to linezolid (100%), erythromycin (50%), ciprofloxacin (75%), clindamycin (100%), cotrimoxazole (75%), and tetracycline (100%). Routine antimicrobial sensitivity of MRSA done by Mahmood et al. showed 28.7% to ciprofloxacin, 37.5% to gentamicin, 35% to clindamycin, 27.5% to erythromycin, 18% to fusidic acid, 8% to penicillin, 87% to moxifloxacin, 0% to oxacillin, 100% to vancomycin, teicoplanin, linezolid, and tigecycline [28]. Relatively high sensitivity towards erythromycin, clindamycin, and ciprofloxacin was seen in our study. The full susceptibility of MRSA towards linezolid, clindamycin, and tetracycline indicates the narrow use of them in the MRSA chemotherapy. However, several reports show MRSA resistant to some of the listed drugs. In a study carried out by Belbase et al., few strains were resistant to tetracycline and clindamycin [29]. MRSA can be eradicated with a prescribed dose of linezolid [30], although treatment protocols differ, and serum levels of antibiotics generally vary from person to person and may affect the outcomes [31].

Macrolide-Lincosamide-Streptogramin B (MLS_B_) has been used to treat skin and soft tissue infection caused by *S*. *aureus*, more often clindamycin, because of its good pharmacokinetic properties leading to the evolution of resistance in many Staphylococci [32]. In the study, out of 11 erythromycin-resistant and clindamycin-sensitive MSSA isolates, 6 (54.5%) showed an inducible clindamycin resistance pattern. Two MRSA that met the criteria were D-test positive. Clindamycin preferably does MRSA infection treatment, but because of the high rate of inducible clindamycin resistance in MRSA and MSSA strains, there exists a high chance of failure showing this type of resistance. Such tests minimize the risk of failure of clindamycin therapy [29].

Two (50%) of the total 4 MRSA isolates and fifteen (88.2%) MSSA isolates out of 17 were *β*-lactamase producers. *β*-lactamase test was done to predict the outcome of susceptibility tests with *β*-lactam antimicrobials. The *β*-lactamase produced hydrolyzes the *β*-lactam ring, hence allowing it to achieve resistance to *β*-lactam drugs [33].

Our study indicated the prevalence of MRSA in the shrines. However, this study was focused on limited areas of Kathmandu valley only, rendering it unsuitable for making an assumption about the direction of transmission of MRSA between primates and humans and estimating accurate MRSA and MSSA persistence in the environment (Tables 5–12).

## 5. Conclusions

MRSA and MSSA carriage rates were 19% and 81%, respectively. All MSSA and MRSA isolates were susceptible to linezolid, tetracycline, and clindamycin; however, high resistance to erythromycin followed by ciprofloxacin in MSSA isolates, and erythromycin and gentamicin in MRSA isolates were observed in the study.

Though the results obtained are concerning and suggest an extensive application of antibiotics, it might not be best to use the results as a sole indicator of antibiotic overuse. Moreover, the use of molecular techniques such as polymerase chain reaction, real-time polymerase chain reaction, or nucleic acid sequence-based amplification in the detection of *Staphylococcus aureus* strains would have exhibited a better result. Further evaluation is essential to understand the extent of MRSA, including shrines from outside of Kathmandu valley.

## Figures and Tables

**Table 1:** Distribution of *S. aureus* among different sites.

Shrines	Total no. of samples	Total no. of *S. aureus*

Pashupati	20	4
Swayambhu	20	3
Thapathali	20	7
Guheshwori	20	2
Nilbarahi	20	4
Bajrayogini	20	1
Grand total		21

**Table 2:** Distribution of MRSA among different sites.

Shrines	Total no. of *S. aureus*	Total no. of MRSA

Pashupati	4	1
Swayambhu	3	0
Thapathali	7	2
Guheshwori	2	0
Nilbarahi	4	1
Bajrayogini	1	0
Grand total		4

**Table 3:** Antibiotic susceptibility pattern of MSSA.

Antibiotics (*μ*g)	Susceptibility patterns		
	Sensitive	Intermediate	Resistance

Erythromycin (15)	6		11
Ciprofloxacin (5)	9		8
Clindamycin (2)	17		
Tetracycline (30)	17		
Cotrimoxazole (1.25/22.75)	13		4
Linezolid (30)	17		
Gentamicin (10)	16		1

**Table 4:** Antibiotic susceptibility pattern of MRSA.

Antibiotics (*μ*g)	Susceptibility patterns		
	Sensitive	Intermediate	Resistance

Erythromycin (15)	2		2
Ciprofloxacin (5)	3		1
Clindamycin (2)	4		
Tetracycline (30)	4		
Cotrimoxazole (1.25/22.75)	3		1
Linezolid (30)	4		
Gentamicin (10)		2	2

**Table 5:** Ingredients used in gram/liter in the preparation of nutrient agar.

Ingredients	Gram/liter
Peptone	5.0
Sodium chloride	5.0
Beef extract	1.5
Yeast extract	1.5
Agar	15.0
PH (at 25°C)	7.4 ± 0.2

About 28 gm of the medium was dissolved in 1000 ml of distilled water and heated to dissolve the media. The media were autoclaved at 15 lbs at 121°C for 15 minutes.

**Table 6:** Amount (in gm/ltr) of ingredients used in the preparation of nutrient broth.

Ingredients	Gram/liter
Peptone	5.0
Sodium chloride	5.0
Beef extract	1.5
Yeast extract	1.5
PH (at 25°C)	7.4 ± 0.2

About 13°gm of the medium was dissolved in 1000 ml of distilled water and heated to dissolve the media. The media were autoclaved at 15°lbs at 121°C for 15 minutes.

**Table 7:** Ingredients used in the preparation of Mannitol salt agar (MSA).

Ingredients	Gram/liter
Proteose peptone	10.0
Sodium chloride	75.0
D-mannitol	10.0
Phenol red	0.025
Agar	15.0
PH (at 25°C)	7.4 ± 0.2

About 111 gm of the medium was suspended in 1000 ml distilled water and heated to dissolve the media. The media were autoclaved at 15 lbs at 121°C for 15 minutes.

**Table 8:** Ingredients in gram/liter used in the preparation of Mueller–Hinton agar (MHA).

Ingredients	Gram/liter

Beef extract	2.0
Casein acid hydrolysate	17.5
Starch	1.5
Agar	17.0
PH (at 25°C)	7.4 ± 0.2

About 38 grams of the medium was suspended in 1000 ml water and boiled to dissolve completely. The media were then autoclaved at 15 lbs at 121°C for 15 minutes.

**Table 9:** Composition of DNase agar used in the study.

Ingredients	Gram/liter

Tryptose	20.0
Deoxyribonucleic acid	2.0
Sodium chloride	5.0
Methyl green	0.0509
Agar	15.0
PH (at 25°C)	7.3 ± 0.2

About 42.05 grams of the medium was dissolved in 1000 ml distilled and boiled to dissolve completely. The media were then autoclaved at 15 lbs at 121°C for 15 minutes.

**Table 10:** Ingredients used in gram/liter in the preparation of M-*Staphylococcus* broth.

Ingredients	Gram/liter
Casein enzymic hydrolysate	10.0
Yeast extract	2.5
Lactose	2.0
Mannitol	10.0
Dipotassium hydrogen phosphate	5.0
Sodium chloride	75.0
Sodium azide	0.049
PH (at 25°C)	7.0 ± 0.2

About 104.55 grams of the media was suspended in 1000 ml distilled water. It was mixed thoroughly and heated to boiling for 5 minutes. The media were then autoclaved at 15 lbs at 121°C for 15 minutes.

**Table 11:** Preparation of catalase reagent.

Hydrogen peroxide	3 ml

Distilled water	100 ml

To 100 ml distilled water, 3 ml of hydrogen peroxide is mixed with 100 ml of distilled water to prepare catalase reagent.

**Table 12:** Preparation of oxidase reagent.

Tetramethyl para-phenylene diamine dihydrochloride	1.0 gm
Distilled water	100 ml

This reagent was made by dissolving 1 g of the reagent in 100 ml of distilled water. To that solution, stripes of Whatman No. 1 filter paper were soaked and drained for about 30 sec. Then, these stripes were completely dried and stored in a dark bottle tightly sealed with a screw cap.

**Table 13:** Zone diameter interpretative standard for *S. aureus*.

Antibiotics	Zone diameter interpretive criteria (nearest whole mm)		
	Sensitive	Intermediate	Resistant

Cefoxitin (30 *µ*g)	≥22	—	≥21
Gentamicin (10 *µ*g)	≥15	13-14	≤12
Erythromycin (15 *µ*g)	≥23	14–22	≤13
Tetracycline (30 *µ*g)	≥19	15–18	≤14
Ciprofloxacin (5 *µ*g)	≥21	16–20	≤15
Clindamycin (2 *µ*g)	≥21	15–20	≤14
Cotrimoxazole (1.25/22.75 *µ*g)	≥16	11–15	≤10
Linezolid (30 *µ*g)	≥21	—	≤20

## Data Availability

No data were used to support this research work.

## Appendix

List of materials and preparation of culture media are as follows:

(A) Reagents/chemicals
    0.5 McFarland standard Antibiotics; Hi-Media; India
    Catalase and oxidase reagent; Hi-Media, India
    Distilled water
    Ethanol; Hi-Media, India
    Gram stain reagents, oxidase reagent discs, H_2_O_2_; Hi-Media, India Human plasma
    NaCl; Hi-Media, India
(B) Glass Wares
    Beaker, glass slides; Borosil Conical flask; Borosil Measuring cylinder; Borosil
(C) Equipment
    Microscope; Olympus, Japan pH meter; Tarsons, India Refrigerator; Sanyo, USA Autoclave Chitransh, India
    Incubator; Associated Scientific Technologist, India Hot air oven; Chitransh, India
(D) Culture Media
    Mannitol Salt Agar (MSA); Hi-Media, India Mueller-Hinton Agar; Hi-Media, India Nutrient agar; Hi-Media, India
    Nutrient Broth; Hi-Media, India DNase agar; Hi-Media, India
    M-Staphylococcus Broth; Difco, USA
(E) Miscellaneous
    Inoculating loop
    Screw-capped cotton wool swab; Hi-Media, India Cotton wool swabs
(F) Preparation of culture media

Composition and preparation of different types of media are as follows.

(G) Composition of different staining and test reagent
  1. Gram staining reagent
  2. Crystal violet solution
  3. Crystal violet stock solution was prepared by dissolving 40 g of crystal violet (90–95% dye content) in 400 ml of ethanol (95%). It was filtered and stored at room temperature. For the preparation of the working solution of crystal violet, 40 ml of stock solution was added to 160 ml of filtered ammonium oxalate solution (1%).
  4. Gram's iodine
  5. To make a stock solution of Lugol's iodine, 25 g of iodine crystals and 50 g of potassium iodide was added in 500 ml of distilled water in a brown glass bottle. For the preparation of a working solution, 60 ml of Lugol's iodine stock solution was added with 220 ml of distilled water and a 60 ml 5% sodium bicarbonate solution.
  6. Safranin
  7. The stock solution of safranin was prepared by dissolving 5 g of safranin in 200 ml of 95% ethanol. For the preparation of a safranin working solution, 20 ml of solution was mixed with 180 ml of distilled water.
  8. acetone alcohol (1 : 1)
  9. 50 ml of ethanol (95%) with 50 ml acetone was mixed in a brown bottle. The date was labelled, and the solution was stored at room temperature.

Biochemical test reagent is as follows:

(1) Turbidity standard equivalent to McFarland 0.5
  - 0.6 ml of 1% barium chloride dehydrate (BaCl_2_·H_4_O_2_) was mixed with 99.4 ml of 1% sulphuric acid.
  - Then, the mixture was mixed completely to form a turbid suspension.
  - A small volume of the turbid solution was transferred into screw-capped tubes of the same size as used for preparing test and control inoculums.
  - The tubes were then stored in the dark and vigorously agitated in a vortex mixture before use.

The standard, when stored in a well-sealed container in the dark at room temperature (20–28°C), may be kept for up to 6 months.

A protocol of Gram staining and biochemical tests.

Gram staining procedure
  - Using a sterile inoculating loop pure isolated colony from nutrient agar was transferred to a clean and grease-free glass slide containing a drop of distilled water.
  - A uniform smear was made on the glass slide, air-dried, and then heat-fixed.
  - The smear was flooded with crystal violet solution for 1 minute and rinsed with distilled water.
  - It was then loaded with Gram's iodine solution for 1 minute and again rinsed with distilled water.
  - The smear was treated with 95% ethanol (decolorizing agent) solution for 10–15 seconds for decolorization and rinsed with distilled water.
  - Finally, the smear was treated with counterstain safranin for 1 minute and rinsed with distilled water.
  - The backside of the slide was blotted with blotting paper, air-dried, and then examined first under 40x objective and then under the oil immersion objective of the microscope.
  - Gram-positive cocci seen in grape-like clusters were indicative of *S. aureus.*
Catalase Test Procedure
  1. A clean and grease-free glass slide was taken. On the slide, a drop of freshly prepared 3% H_2_O_2_ solution was put.
  2. The pure colony from the nutrient agar plate was taken with the help of a sterile glass rod and it was then transferred on the 3% H_2_O_2_ solution.
  3. The rapid evolution of gas bubbles on the slide was indicative of a catalase-positive test.
Oxidase test
  1. By using a sterile glass rod, the isolated colony from the nutrient agar was transferred into the sterile filter paper soaked with 1% tetramethyl para-phenylene diamine dihydrochloride. No change in color was indicative of a negative test.
Coagulase test
  - Slide coagulase (clumping factor/bound coagulase) test procedure
  - A clean and grease-free slide was taken, and a drop of physiological saline was dropped on it.
  - A colony of the test organism was transferred on it with the help of a glass rod, and a thick suspension was made.
  - To the suspension, a drop of plasma was added and mixed gently.
  - Clumping observed within 5–10 seconds was indicative of slide coagulase (clumping factor) positive test. However, both the slide coagulase-positive as well as negative organisms were subjected to the tube coagulase test for further confirmation.
  - Tube coagulase (free coagulase) test procedure
  - Three test tubes were taken and labelled as T (test), P (positive), and N (negative).
  - About 0.2 ml of fresh plasma was pipetted in each test tube.
  - About 0.8 ml of test broth culture was added to the tube labelled as “T,” 0.8 ml of a standard culture of *S. aureus* was added to the one tube labelled as “P,” and 0.8 ml of sterile broth was added to another tube labelled as “N.”
  - After mixing gently, all the tubes were incubated at 37°C and the clot formation was examined at an interval of one hour for up to 4 hours, by tilting the tubes.
  - The negative tubes were kept at room temperature overnight and were reexamined.
  - Stiff gel formation or clot floating in the tube was indicative of a positive tube test. Both in slide and tube coagulase test, MRSA COL strain was used as positive control and *S. aureus* NCTC 8325 as a negative control.
Oxidative and fermentative test
  1. Two tubes of oxidative and fermentative test medium were prepared.
  2. The colony of the test organism was transferred using straight wire by stabbing halfway to the bottom of the tubes.
  3. One tube of each pair was covered with a 1-cm layer of sterile mineral oil or liquid paraffin. Another tube was left open without any covering.
  4. Both tubes were incubated at 35 for 48 hours (slow-growing bacteria may take 3 to 4 days before results can be observed).

Acid production was detected in the medium by the appearance of a yellow color. *S. aureus* was detected by acid production on both (open and covered) tubes. The acid produced changed the pH indicator, bromothymol blue, from green to yellow.

DNase test
  1. With the help of a sterile loop, colonies from 18 to 24 hours were picked and inoculated onto a small area of the DNase test agar plate. *S. aureus* ATCC was used as positive control and *E. coli* ATCC 25922 as a negative control.
  2. The plate was incubated at 37°C for 18–24 hours.
  3. After incubation, the surface of agar was flooded with 1 N HCL solution, and excess acid was removed.
  4. The clear zone around the colonies was observed within five minutes, which indicated the positive test.

Zone diameter/MIC interpretive criteria for *S. aureus* in Table 13.
